# Combined Interventional Radiological and Endoscopical Approach for the Treatment of a Postoperative Biliary Stricture and Fistula

**DOI:** 10.1155/1995/48252

**Published:** 1995

**Authors:** Jürgen Triller, Adrian Schmassmann, Walter Schweizer, Abraham Czerniak

**Affiliations:** 1 From the Institute for Diagnostic Radiology University Inselspital of Berne Berne CH-3010 Switzerland; 2 Division of Gastroenterology University Inselspital of Berne Berne CH-3010 Switzerland; 3 Department of Visceral and Transplantation Surgery University Inselspital of Berne Berne CH-3010 Switzerland

## Abstract

A 43-year old woman was admitted 11 days after open cholecystectomy with a iatrogenic bile duct injury. On admission the patient showed an uncontrolled biliary fistula through an external drain placed at an emergency laparotomy for biliary peritonitis with fever and jaundice. PTC showed a biliary stricture type II (Bismuth). A percutaneous drainage was performed to decompress the biliary system. Three weeks later, percutaneous balloon dilatation of the stricture was performed. However, bile leakage persisted. In a combined transhepatic/ endoscopic procedure, the percutaneous biliary drainage was replaced by a nasobiliary tube. One week later, no stricture was found and the biliary leak was sealed. The patient could be discharged without symptoms or signs of cholestasis. The multidisciplinary management of post-operative biliary fistula is presented, comparing the role of interventional radiology, endoscopy and surgery.


*HPB Surgery*, 1995, Vol. 8, pp. 257–262
Reprints available directly from the publisher
Photocopying permitted by license only

© 1995 OPA (Overseas Publishers Association)
Amsterdam B.V. Published in The Netherlands
by Harwood Academic Publishers GmbH
Printed in Malaysia

### Combined Interventional Radiological and Endoscopical Approach for the Treatment of a Postoperative Biliary Stricture and Fistula

JÜRGEN TRILLER[1], ADRIAN SCHMASSMANN[2], WALTER SCHWEIZER[3], and ABRAHAM CZERNIAK[3]

[1]From the Institute for Diagnostic Radiology, [2]Division of Gastroenterology, [3]Department of Visceral and Transplantation Surgery, University Inselspital of Berne CH-3010 Berne, Switzerland

*(Received August 10, 1994)*

A 43-year old woman was admitted 11 days after open cholecystectomy with a iatrogenic bile duct injury. On admission the patient showed an uncontrolled biliary fistula through an external drain placed at an emergency laparotomy for biliary peritonitis with fever and jaundice. PTC showed a biliary stricture type II (Bismuth). A percutaneous drainage was performed to decompress the biliary system. Three weeks later, percutaneous balloon dilatation of the stricture was performed. However, bile leakage persisted. In a combined transhepatic/endoscopic procedure, the percutaneous biliary drainage was replaced by a nasobiliary tube. One week later, no stricture was found and the biliary leak was sealed. The patient could be discharged without symptoms or signs of cholestasis. The multidisciplinary management of post-operative biliary fistula is presented, comparing the role of interventional radiology, endoscopy and surgery.

KEY WORDS: Cholecystectomy biliary fistula interventional radiology endoscopy surgery

### INTRODUCTION

Iatrogenic bile duct injuries occur in 0.2 to 0.5% of patients during cholecystectomy[1]. Treatment of these usually mid-common hepatic duct injuries is aimed at restoring continuity of biliary flow [2]. Operative repair has a long-term recurrence rate of 10 to 30% in specialized centers[3], although some series quote lower values[4]. Operative mortality is reported as being between 5 and 8%[5] but may reach 13%[2].

Non-operative alternatives have therefore been developed. Both percutaneous[6] and endoscopic biliary drainage procedures[7] in combination with balloon dilatation or stent placement[8] have been attempted with variable long-term success rates. Non-randomized studies suggest that the non-surgical approach is an acceptable alternative for the treatment of benign biliary strictures [8–10].

While endoscopic and transhepatic approaches have been successful, both methods have inherent limitations and in about 10 to 15% a combined procedure is necessary for diagnosis and therapy[11, 12]. The present case describes a combined transhepatic/endoscopic procedure including percutaneous transhepatic biliary drainage, balloon dilatation and nasobiliary drainage in a patient with postoperative biliary stricture and fistula.

### CASE REPORT

A 43-year old woman with multiple symptomatic gallstones had an open cholecystectomy in a district hospital. During the operation the common hepatic duct was

*Address for correspondence:* W. Schweizer, M.D. Department of Visceral and Transplantation Surgery, University Inselspital of Berne, CH-3010 Berne Switzerland.

injured because of adhesions and chronic inflammation in the region of the Calot's triangle. The common hepatic duct leak was oversewn. No operative cholangiography was performed and no T-tube placed into the bile duct. During the following days, the patient developed jaundice and fever which were treated with antibiotics. One week later, an intra-abdominal bile collection with biliary peritonitis was externally drained by a second operation. Because of progressive jaundice the patient was referred to our unit eleven days after the initial operation.

On admission, the patient was jaundiced and in moderate general condition. Laboratory results revealed a cholestatic pattern: bilirubin: 162 $\mu$mol/l (normal: 3.4–25.7 $\mu$mol/l), alkaline phosphatase: 507U/l (normal 36–108 U/l). Antibiotic therapy with ceftriaxon was continued. CT revealed left pleural effusion, perihepatic fluid collection and dilated intrahepatic bile ducts with no dilatation of the distal common bile duct. HIDA scanning showed retention of the tracer in the liver suggesting high-grade obstruction of the common bile duct.

ERCP allowed visualization of the pancreatic duct only; the bile duct could neither be cannulated nor visualized. PTC showed a high-grade stricture in the common hepatic duct, 2 cm below the bifurcation, classified as Type 2 stricture according to the Bismuth classification (Figure 1)[5]. Once biliary anatomy had been delineated, the bile duct of the liver segment VII was repunctured using a 5-French Accustic Set (Meditech). Then, a guide-wire was placed into the biliary tree and finally a 8.3-French Ringg drainage catheter (Cook) was placed above the stricture to decompress the obstructed biliary system. After injection of contrast medium there was evidence of a biliary fistula (Figure 2). Bile flow through the external catheter was 200–500 ml/day. The bilirubin decreased and reached normal limits after 8 days.

Six days after placement of the percutaneous transhepatic biliary drainage, percutaneous balloon dilatation with a 6-French PTA catheter (Guerbet Company) was performed (length 4 cm, diameter 6 mm, pressure 6 atm, 3 x20 sec.) (Figure 3). After balloon dilatation, no stricture could be demonstrated. The biliary fistula from the common hepatic duct into the perihepatic region could still be demonstrated. An 8.3-French multiple-side-hole Ringg drainage catheter (Cook) was placed with its tip in the duodenum. Side holes were placed on both sides of the fistula tract, allowing internal and external drainage of bile.

Two days later, a combined radiologic/endoscopic procedure was performed. An Olympus side-view duodenoscope (JF-100) was placed in front of the papilla of Vater. A guide-wire (0.021 inch, length 4 m) was pushed through the external biliary catheter into the duodenum. The guide-wire was caught with a polyp snare. While the tip of the percutaneous catheter was still in the duodenum, the guide-wire was pushed through the catheter and pulled through the instrumentation channel until one end of the guide-wire was on

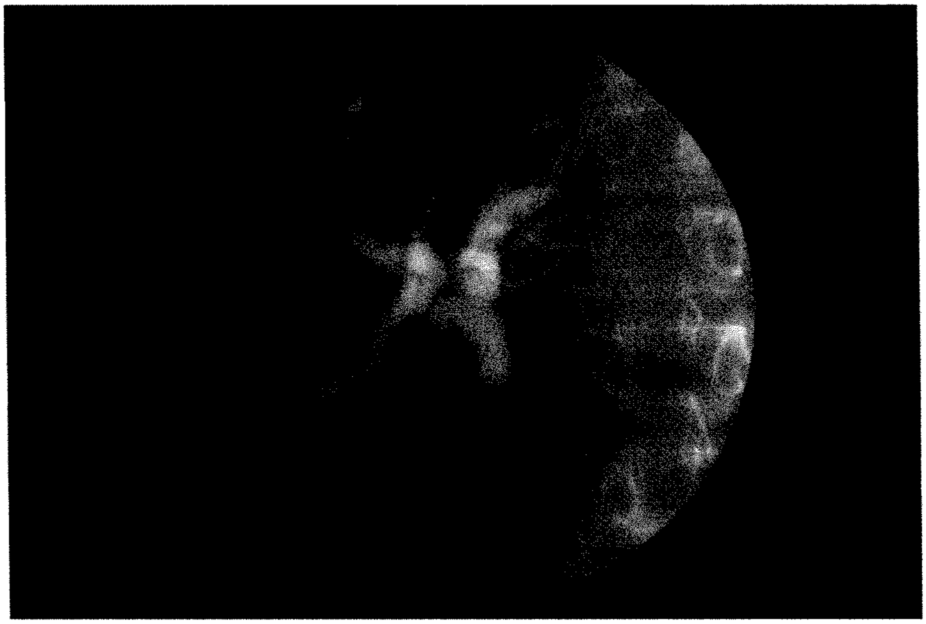

**Figure 1** Percutaneous transhepatic cholangiography showing Bismuth type 2 stricture.

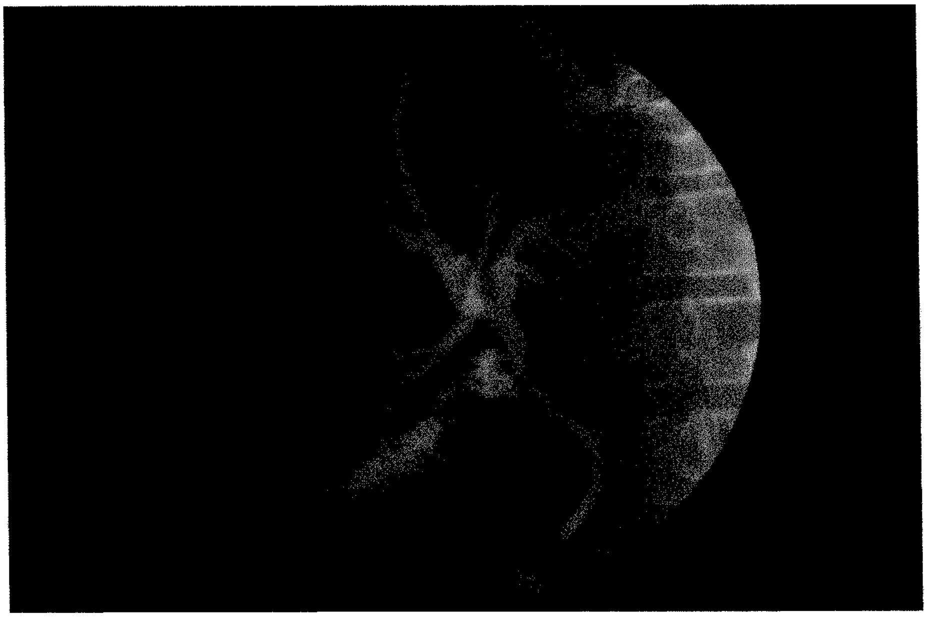

**Figure 2** Percutaneous transhepatic drainage demonstrating a biliary fistula at the side of the stricture.

the radiological and the other on the gastroenterological side. A wire-guided papillotome (Olympus) was properly placed into the distal bile duct and a small sphincterotomy was performed. While the percutaneous catheter was removed, a 9-French nasobiliary tube was pushed over the guide-wire into the right hepatic duct. The guide-wire was removed and contrast medium was injected through the nasobiliary tube. The common hepatic duct was completely normal with no signs of restricture. However, the biliary leakage from the hepatic duct into the perihepatic region was still present (Figure 4).

During the following days bile flow through the nasobiliary tube was 200–500 ml/day and cholestatic

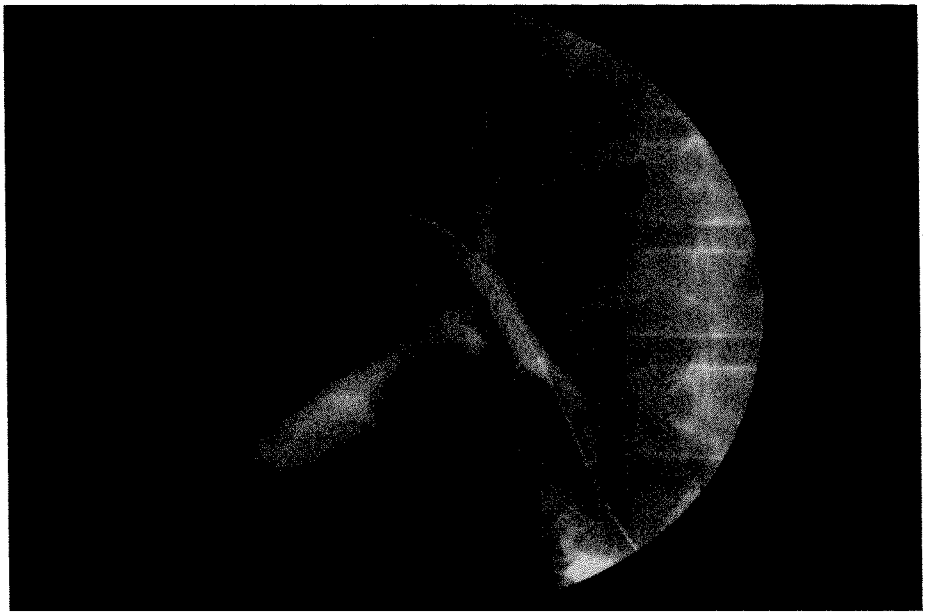

**Figure 3** Transhepatic balloon dilatation.

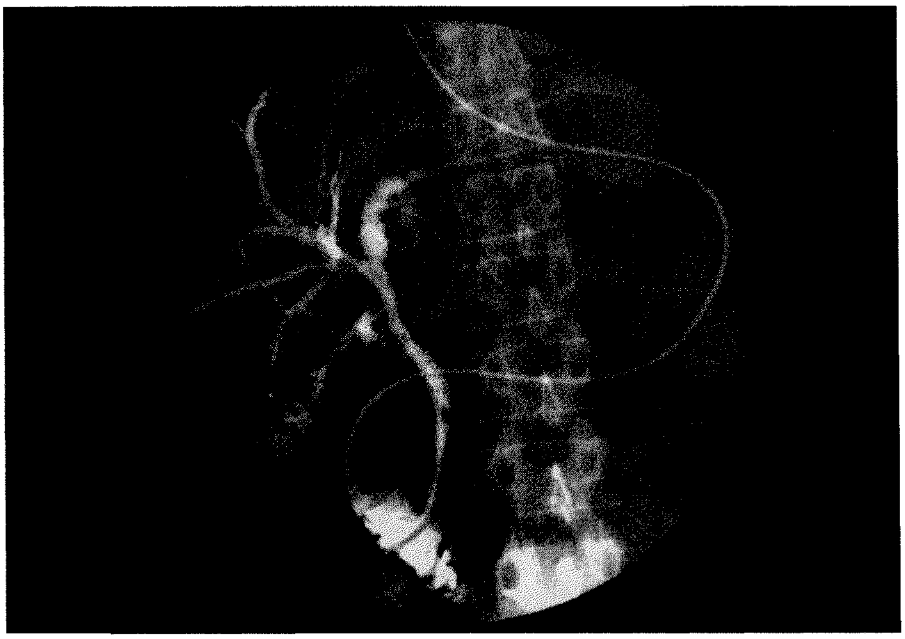

**Figure 4** Cholangiography through the nasobiliary drainage showing the dilated stricture with persistent fistula.

enzymes normalized. The abdominal drainage could be removed and the patient became asymptomatic. One week later, a cholangiogram was performed through the nasobiliary tube. No restricture could be found. The fistula was closed and normal contrast medium emptying into the duodenum was seen (Figure 5). The nasobiliary tube was removed and the patient could be discharged the following day. At follow up after 12 months, no enzymatic or clinical signs for restricturing could be found.

### DISCUSSION

Postoperative biliary strictures, if improperly treated, can lead to high morbidity and mortality[4, 5, 17]. Only about a third of the iatrogenically induced ductal

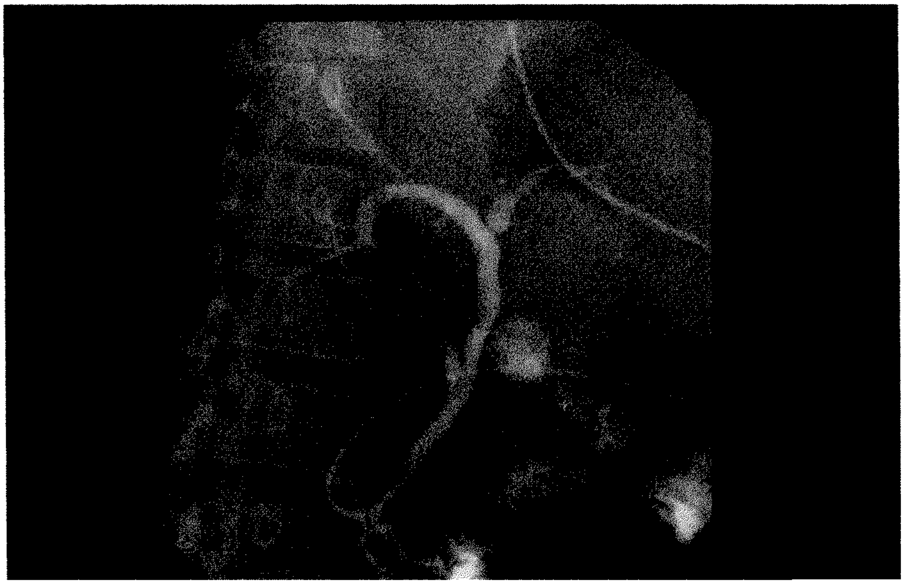

**Figure 5** Cholanigiography after closure of the fistula with totally patent bile ducts.

injuries are recognized in the postoperative period[17]. Clinical presentation may include jaundice, cholangitis and uncontrolled biliary fistula with peritonitis[2,4,13].

ERCP allows a proper diagnosis of post-operative biliary structure in >90% of patients [8,14]. In our hospital, ERCP is therefore the first diagnostic procedure, especially in patients with biliary obstruction in the distal and mid common hepatic duct. ERCP allows-if indicated-sphincterotomy, nasobiliary drainage, balloon dilation and stenting[7,8,14]. PTC/PTCD is indicated, if bile duct cannulation cannot be achieved, if a complete obstruction of the bile duct is found or if the whole intrahepatic biliary tree cannot be properly visualized [5].

Patients with iatrogenic bile duct injuries, uncontrolled biliary fistula and sepsis are mainly treated conservatively today. The therapeutic principle of the management of a biliary stricture with a biliary fistula is to relieve the obstruction, to establish a free bile flow and to allow the inflammatory reaction to decrease. Once this is achieved – the fistula will usually close. Percutaneous bile duct drainage allows a conservative management of critical patients over a long period of time[10, 13]. Following these therapeutical measures, cholangitis usually decreases after a few days. After placement of an external bile drainage with establishment of a controlled fistula, further treatment depends on the extent of the bile duct injury. If the duct is fully dissected or completely obstructed, early surgery is necessary. If cholangiography shows a low-grade stricture, conservative treatment is usually sufficient.

In our patient a high-grade iatrogenic stricture in the common hepatic duct was present. Since the stricture could be passed with a guide-wire, it is possible to treat the fistula and the stricture with one of the following interventional techniques: PTCD with internal-external drainage, dilatation, placement of a naso-biliary tube or implantation of an endoprosthesis [8,9,15].

Balloon dilatation allows temporary or definitive treatment of a postoperative bile duct stricture[10,16,17]. The best results are achieved in patients with unilocular postoperative strictures[18]. Several dilatations can be performed during the drainage procedure. The three year results after percutaneous dilatation of postoperative biliary strictures have shown a success rate of 75% [15].

In our patient, the transhepatic placement of an external biliary drainage was performed after localisation of the stricture and evidence of the fistula using PTC. Percutaneous bile duct dilatation leads to a complete widening of the stricture. To spare the patient from the disadvantages of a long-term percutaneous drainage, the external drainage was replaced by an internal drainage with a naso-biliary tube. After this therapy a complete decompression of the bile duct system occurred with closing of the fistula, relief of jaundice and cholangitis.

The decision for a conservative procedure after successful dilatation requires a long-term follow-up to establish recurrences early. Should there be evidence of a recurrence, the stricture can be redilated or a surgical procedure employed. Comparable studies by Pitt *et al.* [19] have shown a success rate of 88% (after 57 months) following surgery and 55% (after 59 months) following dilatation. Repeated dilatation should therefore only be employed if the risks of a surgical procedure are deemed to be too high[19].

Further reduction of the restricturing rate may be possible by the use of biliary stents, although the indication for stenting in patients with benign disease is controversial[20-22]. Short-term stenting is employed today in order to avoid early restricture after dilatation of a stenosis, to support the closure of the fistula as well as to spare the patient from the inconvenience of an external bile drainage. Long-term stenting may lead to a reduction of late recurrences[17]. However, the advantage of achieving a permanent dilatation of the stricture by means of placing endoprostheses (10–12F) has disadvantages as well. A long-term implantation of an endoprosthesis (6–12 months) induces fibrotic changes which can negatively influence a later surgical procedure[15, 18].

If dilatation leads to relief of the stricture and of the clinical symptoms, surgery may be postponed until there is a recurrence. The surgical reconstruction of the bile ducts can be carried out under optimal conditions in the event of further biliary obstruction. In complex situations, the combined surgical-interventional-radiological procedure using the Roux-Y loop subcutaneously for possible later radiological dilatations may be favorable[23]. Since the clinical symptoms of an incomplete biliary stricture may be obscured, it is necessary that patients undergo regular clinical checkups over a longer period of time (up to 5 years).

With the gradual procedure described here, individual treatment of postoperative bile duct strictures and fistulas may adequately be achieved, depending on the long-term follow up. Patients may be treated at the time with or without the aid of surgery.

### REFERENCES

1. Michie, W., Gunn, A. (1964) Bile duct injuries: a new suggestion for their repair. *British Journal of Surgery*, **51**, 96–100.

2. Warren, K.W., Mountain, J.C., Midell, A.I. (1971) Management of strictures of the biliary tract. *Surgical Clinics of North America*, **51**, 711–731.
3. Pitt, H.A., Miyamato, T., Parapatis, S.K., Tompkins, R.K., Longmire, W.P. (1982) Factors influencing outcome in patients with postoperative biliary strictures. *American Journal of Surgery*, **144**, 14–21.
4. Blumgart, L.H., Kelly, C.J., Benjamin, I.S. (1980) Benign bile duct strictures following cholecystectomy: critical factors in management. *British Journal of Surgery*, **71**, 836–843.
5. Blumgart, L.H. (1988) Benign biliary strictures. In: L.H. Blumgart (ed) Surgery of the Liver and Biliary Tract. Vol. 1. pp. 721–752, Churchill Livingstone, London.
6. Toufanian, A., Carey, L.C., Martin, E.T. Jr. (1978) Transhepatic biliary dilatation: an alternative to surgical reconstruction. *Current Surgery*, **35**, 70–73.
7. Kozarek, R.A. (1986) Hydrostatic balloon dilation of gastrointestinal stenoses: a national survery. *Gastrointestinal Endoscopy*, **32**, 15–19.
8. Huibregtse, K., Cheng, J., Coene, P.P.L.O., Fockens P., Tytgat. G.N.J. (1989) Endoscopic placement of expandable metal stents for biliary strictures a– preliminary report on experience with 33 patients. *Endoscopy*, **21**, 280–282.
9. Miller, T.A. (1990) Biliary stricture: is dilatation an acceptable alternative to operation? *Gastroenterology*, **98**, 1089–1090.
10. Kaufman, S.L., Kadir, S., Mitchell, S.E., Chang, R., Kinnison. M.L., Cameron, J.L., White, R.I. Jr. (1985) Percutaneous transhepatic biliary drainage for bile leaks and fistulas. *American Journal of Roentgenology*, **144**, 1055–1058.
11. Mason, R.R., Cotton, P.E. (1981) Combined duodenoscopic and transhepatic approach to stenosis of the papilla of Vater. *British Journal of Radiology*, **54**, 678–69.
12. Chespal, L.W., Ring, E.J., Shapiro, H.W., Gordon, R.L., Ostoff, J.W. (1989) Multidisciplinary approach to complex endoscopic biliary intervention. *Radiology*, **1770**, 995–997.
13. Kune, G.A., Blumgart, L.H. (1988) External biliary fistula. In: L.H. Blumgart (ed) Surgery of the Liver and Biliary Tract. Vol. 1. pp. 765–789, Churchill Livingstone, London.
14. Cotton, P.B. (1990) Critical appraisal of therapeutic endoscopy in bilary tract diseases. *Annual Review of Medicine*, **41**, 211–222.
15. Mueller, P.R., vanSonnenberg, E., Ferrucci, J.T. Jr., Weyman, P.J., Butch, R.J. Malt, R.A., Burhenne, H.J. (1986) Biliary dilatation: multicenter review of clinical management in 73 patients. *Radiology*, **160**, 17–22.
16. Vogel, S.B., Howard, R.J., Caridi, J., Hawkins, I.F. Jr. (1985) Evaluation of percutaneous transhepatic balloon dilatation of benign biliary strictures in high risk patients. *American Journal of Surgery*, **149**, 73–79.
17. Williams, H.J. Jr., Bender, C.E., May, G.R. (1987) Benign postoperative biliary strictures: dilatation with fluoroscopic guidance. *Radiology*, **163**, 629–634.
18. Morrison, M.C., Lee, M.J., Saini, S., Brink, J.A., Mueller, P.R. (1990) Percutaneous balloon dilation of benign biliary strictures. *Radiologic Clinics of North America*, **28**, 1191–1201.
19. Pitt, H.A., Kaufman, S.L., Coleman, J., *et. al.* (1989) Benign postoperative biliary strictures: Operate or dilate? *Annals of Surgery*, **210**, 417–425.
20. Geenen, D.J., Geenen, J.E., Hogan, W.J., Schenck, J., Venu. R.P., Johnson. G.K., Jackson. A. Jr. (1989) Endoscopic therapy for benign bile duct strictures. *Gastrointestinal Endoscopy*, **35**, 367–371.
21. David, P.H.P., Rauws, E.A.J., Coene, P.P.L.O., Tytgat, G.N.J., Huibregtse, K. (1992) Endoscopic stenting for post-operative biliary strictures. *Gastrointestinal Endoscopy*, **38**,12–18.
22. Gallacher, D.J., Kadir, S., Kaufman, S.T., *et al.* (1985) Nonoperative management of benign postoperative biliary strictures. *Radiology*, **156**, 624–629.
23. Schweizer, W.P., Matthews, J.B. Baer, H.U., Nudelmann, L.I., Triller, J., Halter, F., Gertsch, Ph., Blumgart, L.H. (1991) Combined surgical and interventional radiological approach for complex benign biliary tract obstruction. *British Journal of Surgery*, **78**, 559–5563.

